# Whitefly (*Bemisia tabaci*) genome project: analysis of sequenced clones from egg, instar, and adult (viruliferous and non-viruliferous) cDNA libraries

**DOI:** 10.1186/1471-2164-7-79

**Published:** 2006-04-11

**Authors:** Dena Leshkowitz, Shirley Gazit, Eli Reuveni, Murad Ghanim, Henryk Czosnek, Cindy McKenzie, Robert L Shatters, Judith K Brown

**Affiliations:** 1 The Hebrew University Bioinformatics Unit, The Hebrew University of Jerusalem, Rehovot 76100, Israel; 2 The Robert H Smith Institute for Plant Science and Genetics in Agriculture, The Hebrew University of Jerusalem, Rehovot 76100, Israel; 3 Mouse biology Programme, EMBL, Monterondo, Roma 00016, Italy; 4 Institute of Plant Protection, Department of Entomology, Volcani Center, Bet Dagan 50250, Israel; 5 USDA-ARS U.S. Horticultural Research Laboratory, Fort Pierce, FL 34945, USA; 6 Department of Plant Sciences, University of Arizona, Tucson, AZ 85721, USA

## Abstract

**Background:**

The past three decades have witnessed a dramatic increase in interest in the whitefly *Bemisia tabaci*, owing to its nature as a taxonomically cryptic species, the damage it causes to a large number of herbaceous plants because of its specialized feeding in the phloem, and to its ability to serve as a vector of plant viruses. Among the most important plant viruses to be transmitted by *B. tabaci *are those in the genus *Begomovirus *(family, Geminiviridae). Surprisingly, little is known about the genome of this whitefly. The haploid genome size for male *B. tabaci *has been estimated to be approximately one billion bp by flow cytometry analysis, about five times the size of the fruitfly *Drosophila melanogaster*. The genes involved in whitefly development, in host range plasticity, and in begomovirus vector specificity and competency, are unknown.

**Results:**

To address this general shortage of genomic sequence information, we have constructed three cDNA libraries from non-viruliferous whiteflies (eggs, immature instars, and adults) and two from adult insects that fed on tomato plants infected by two geminiviruses: *Tomato yellow leaf curl virus *(TYLCV) and *Tomato mottle virus *(ToMoV). In total, the sequence of 18,976 clones was determined. After quality control, and removal of 5,542 clones of mitochondrial origin 9,110 sequences remained which included 3,843 singletons and 1,017 contigs. Comparisons with public databases indicated that the libraries contained genes involved in cellular and developmental processes. In addition, approximately 1,000 bases aligned with the genome of the *B. tabaci *endosymbiotic bacterium *Candidatus *Portiera aleyrodidarum, originating primarily from the egg and instar libraries. Apart from the mitochondrial sequences, the longest and most abundant sequence encodes vitellogenin, which originated from whitefly adult libraries, indicating that much of the gene expression in this insect is directed toward the production of eggs.

**Conclusion:**

This is the first functional genomics project involving a hemipteran (Homopteran) insect from the subtropics/tropics. The *B. tabaci *sequence database now provides an important tool to initiate identification of whitefly genes involved in development, behaviour, and *B. tabaci*-mediated begomovirus transmission.

## Background

The past three decades have witnessed a dramatic increase in the economic importance of the whitefly *Bemisia tabaci *(Genn.) (Aleyrodidae; Hemiptera) in subtropical and mild temperate agriculture systems, owing to the damage it causes to plants when it feeds in the phloem, and its ability to transmit plant viruses. *B. tabaci *occupies tropical and subtropical habitats, producing 11–15 generations per year [1,2]. The *B. tabaci *complex consists of diverse biological 'types' with distinct genetic polymorphisms [3,4], and differences in host range, fecundity, dispersal behaviours, prokaryotic endosymbiont composition, and competency with respect to begomovirus transmission, a group of small circular ssDNA plant viruses (genus *Begomovirus*, family *Geminiviridae*) [5,6]. The highly fecund Old World B biotype can produce ~ 300 eggs/female, colonizes over 500 host species, while the New World A type colonizes about 200 species and has a lower fecundity (~ 100 eggs/female). In contrast, the Jatropha type colonizes only a few species within the genus *Jatropha *and exhibits low fecundity (~ 30–50 eggs/female) [3].

*B. tabaci *adults develop from eggs, after passing through four instars in approximately 2–3 wk and development is temperature dependent. Members of this complex are haplodiploid and thus unfertilized eggs give rise to haploid males; fertilized eggs develop into diploid females (arrhenotoky) [1,2].

The B type of *B. tabaci *transmits begomoviruses to a large number of crop, ornamental, and weed species [7]. Begomovirus have either one (monopartite) or two (bipartite) genomic components [8]. Those infecting tomato constitute a large group of begomoviruses. Among them the bipartite *Tomato mottle virus *(ToMoV) originated in the New World (Florida/Caribbean region), whereas, the monopartite Old World *Tomato yellow leaf curl virus *(TYLCV) is indigenous to the Old World (Middle East and Africa). TYLCV recently was introduced to the Caribbean Islands and has since spread into the South eastern states of the U.S.A. [9].

Begomoviruses are transmitted by *B. tabaci *in a circulative manner [10,11]. Virus particles ingested through the stylets enter the oesophagus and the filter chamber, are transported through the gut into the hemocoel, reach the salivary glands and are finally 'transmitted' during feeding, about 8–12 h after the beginning of an acquisition access period [10]. Velocity of translocation is reported to constitute an intrinsic property of the vector, not of the virus [12,13]. *B. tabaci *is able to transmit begomoviruses, and in particular TYLCV, for its lifetime, after the latent period has been achieved [14,15]. The ingestion of TYLCV by the whitefly vector is accompanied by a marked decrease in whitefly longevity and fertility [15]. In contrast whiteflies that have ingested ToMoV displayed higher fecundity when reared on virus-free tomato than whiteflies not exposed to the virus [16]. TYLCV transcripts have been found in *B. tabaci *harbouring this virus, whereas viral transcripts are not detected in whiteflies that have ingested ToMoV [17], suggesting a fundamental difference in interactions between these two begomoviruses and their whitefly vector.

At least one whitefly species that colonizes some of the same hosts as *B. tabaci *(e.g. the greenhouse whitefly, *Trialeurodes vaporariorum*) is known to be capable of ingesting, but does not transmit begomoviruses [12], and at least one barrier to transmission has been shown to occur at the gut/hemocoel interface [12,18]. The receptors that are hypothesized to mediate begomovirus translocation into the salivary glands of *B. tabaci*, which is a requisite to transmission, and their genes, are presently unidentified.

Surprisingly very little is known about the genetic make up of this insect. The nuclear DNA content of *B. tabaci *male and female was estimated as 1.04 and 2.06 pg respectively, using flow cytometry, indicating that the haploid genome of *B. tabaci *contains about one billion bp, which is approximately five times the size of the genome of the fruitfly *Drosophila melanogaster *[19]. However, it is still not clear if this size estimate will prove to be accurate and so a long-term goal is to determine the complete genome of this whitefly. Ultimately it is of interest to isolate and identify the genes expressed during the life cycle of the whitefly *B. tabaci *and to understand the genetic makeup of this pest. Of particular interest is the identification of specific genes and their functions, which are expressed during the development of *B. tabaci*, as well as those involved in circulative virus transmission, the detoxification of insecticides, and the determination of polyphagy or monophagy in different *B. tabaci *biotypes. Consequently, the construction of cDNA libraries and the analyses of the sequences for the widespread 'B' biotype of *B. tabaci *constitute a first step in this endeavour.

## Results

### Preparation of cDNA libraries

Five independent cDNA, or expressed sequence tag (EST), libraries were prepared from mRNA isolated from adult *B. tabaci *'B' biotype whiteflies: (1) reared in Israel on cotton plants [a non-host of TYLCV/ToMoV (= HBT)], or reared in Florida, USA on (2) TYLCV-infected tomato plants (= TYLCV), and (3) ToMoV-infected tomato plants (= TOMOV). Libraries also were constructed from mRNA isolated from the eggs of non-viruliferous whiteflies (= EGG), and immature instars (crawler to pupae) of non-viruliferous insects (= INST). The libraries were not normalized or amplified.  Among them the bipartite Tomato mottle virus (ToMoV) originated in the New World (Florida/Caribbean region), whereas, the monopartite Tomato yellow leaf curl virus (TYLCV) is indigenous to the Old World (Middle East and Africa).

### Assessment of library and sequence quality

From 18,976 sequencing attempts 9,110 sequences remained after quality, vector and adapter trimming, and removal of mitochondrial DNA sequences (see Materials and methods). The fraction of cleaned sequences from the total number of sequences from each library was between one half and one third (Table 1). The number of sequences from the various libraries that were assembled into contigs and singletons was as follows: EGG: 201, INST: 1816, HBT: 2093, TYLCV: 2704, and TOMOV: 2296 (Table 1).

**Table 1:** Number of sequences from the various libraries and number of sequences assembled into contigs (sequences of mitochondrial origin were removed from the contig assembly process)

	**Number of sequences**	**From EGG**	**From INST**	**From HBT**	**FromTYLCV**	**From TOMOV**
Total number of sequenced clones	18,976	673	3,745	4,321	5,857	4,380
Clones of mitochondrial origin	5,542	59	866	1,576	1,465	1,576
Sequences in contigs and singletons	9,110	201	1,816	2,093	2,704	2,296

### EST assembly into contigs

**Figure 1:**
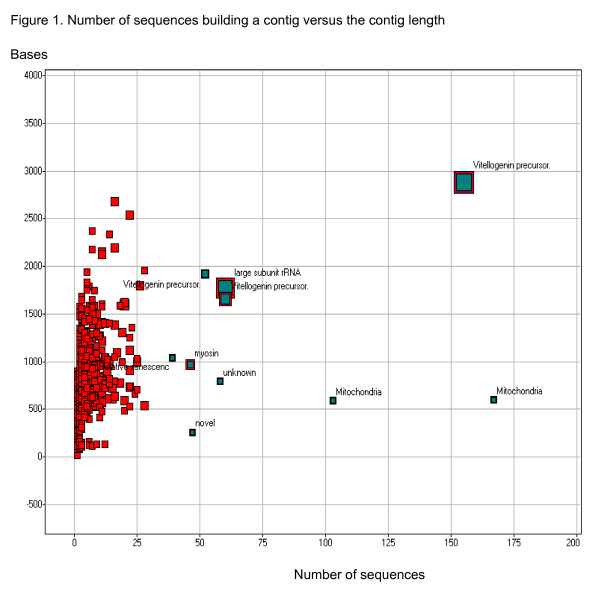
**Number of sequences building a contig versus the contig length **Scatter plot of contigs sequence number (x axis; number of sequences that make up a certain contig) versus the contig length (y axis; bases). The colour scale represents the amount of sequences; the size of the square represents the number of HBT sequences. The annotations for the ten contigs, having the highest number of sequences is shown.

**Figure 2:**
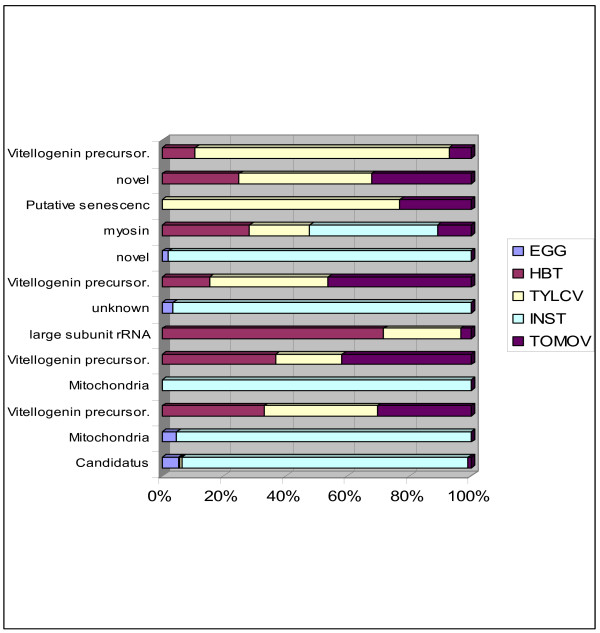
**Library distribution for the most abundant contigs **The figure represents for each of the 13 most abundant contigs as revealed by their sequence composition. The number of sequences building the contigs is from 28 to 425. The percentage in based on the total number of sequence forming the contig. The contigs are represented by their annotation.

To identify clones belonging to the same gene, sequences were assembled into contigs [see Additional files 1 and 2] using the Staden Gap4 program [20]. The advantage of this program is that both the bases and their quality are used to assess overlap and contig consensus sequence. In the assembly process the genome of the whitefly primary endosymbiotic bacteria *Candidatus *Portiera aleyrodidarum [21] (AY268081.1) was included since a preliminary analysis revealed that some of the sequences were identical to bacterial DNA. In the assembly process 4,860 contigs and singletons were assembled from 9,109 sequences (Table 2). The number of singletons was 3,843. The GC content and the average length were higher in the contigs than in the singletons. The contigs with more than a single sequence resulted in sequences 1.5 fold longer than the average sequence length. Figure 1 shows that the contigs with up to 25 sequences had a linear relationship between sequence number and contig length, i.e. the assembly process produces longer sequences than the singletons. Table 3 shows that the largest contig (with respect to sequence number) was assembled with sequences from the genome of *Candidatus *Portiera aleyrodidarum (AY268081.1), and was derived primarily from egg (EGG) and instar (INST) libraries; endosymbiotic sequences were rare in adult whitefly libraries (Figure 2). Most clones that shared sequence homology with this bacterium aligned between nucleotide coordinates 23,000–24,000 of the partial sequence of the bacterial genome, which encodes a 16S ribosomal RNA gene. The region downstream was found to be rich in adenine, a feature which may have contributed to poly(T)-mediated capture intended to selectively bind poly(A)-containing eukaryotic mRNAs.

**Table 2:** Contig and singleton statistics. Breakdown with respect to their number. GC content, average length and % annotated are shown. Annotation was determined by having a homolog in any of the databases searched with an E-value of 1.0e-06. Even though the *Candidatus *Portiera aleyrodidarum bacterial DNA was present in the assembly processes, the sequences were removed when statistical calculations were carried out (represented by *) to avoid distortion of the results

	**Number of clones**	**Average GC**	**Average Length**	**Annotated**
All sequences	4,860	0.34*	515*	45.5%
Number of contigs	1,017	0.38*	785.5*	58%
Number of singletons	3,843	0.34	443	42%

*excluding sequences from the endosymbiont *Candidatus *Portiera aleyrodidarum

**Table 3:** Information on the most abundant contigs. The number of sequences that compose the contigs and the source library, their length, GC content and annotation are indicated. The contig with the highest number of sequences was identified as part of the *B. tabaci *primary symbiont, *Candidatus *Portiera aleyrodidarum, based on a partial genomic sequence (gi|32423678|gb|AY268081.1|). This DNA sequence was included in the assembly process. Among the most the abundant contigs were *B. tabaci *mitochondrial genome sequences (most of them were removed during the preassembly stage)

**Contig name**	**Number of sequences**	**Length (bp)**	**GC content**	**EGG**	**INST**	**HBT**	**TYLCV**	**TOMOV**	**Annotation**
*Candidatus*	425	31,123*	0.3	22	393	3	2	4	*Candidatus*
Bt-HInst-045-1-B11-T3_B11	167	598	0.16	8	159	0	0	0	Mitochondria
Bt_TYLCV004_B07	155	2,883	0.48	0	0	51	57	47	Vitellogenin precursor
Bt-HInst-008-1-D2-T3_D02	103	589	0.15	0	103	0	0	0	Mitochondria
Bt-ToMoV-020-1-D2-T3_D02	60	1,660	0.64	0	0	22	13	25	Vitellogenin precursor
Bt-H-024-1-D9-T3_D09	60	1,775	0.47	0	0	43	15	2	Large subunit rRNA
Bt-HInst-032-1-E6-T3_E06	58	792	0.46	2	56	0	0	0	Unknown
Bt-TYLCV-043-1-D2-T3_D02	52	1,922	0.46	0	0	8	20	24	Vitellogenin precursor
Bt-HInst-013-1-H10-T3_H10	47	260	0.38	1	46	0	0	0	Novel
Bt-HInst-003-1-G5-T3_G05	46	970	0.4	0	19	13	9	5	Myosin
Bt-ToMoV-034-1-B10-T3_B10	39	1,039	0.65	0	0	0	30	9	Putative senescence
Bt-ToMoV-023-1-C12-T3_C12	28	542	0.46	0	0	7	12	9	Novel (signal peptide & transmembranal domain)
Bt-TYLCV-030-1-C9-T3_C09	28	1,959	0.44	0	0	3	23	2	Vitellogenin precursor

* In the assembly process the sequence of the whitefly primary endosymbiotic bacteria *Candidatus *Portiera aleyrodidarum [21] (AY268081.1) was included (see Results, EST assembly into contigs)

The second largest contig was composed of sequences homologous to the published *B. tabaci *mitochondrial genome (AY521259). The number of mitochondrial DNA clones was extremely high. We performed an initial screening for mitochondrial sequences by running RepeatMasker against the published mitochondrial genome (see Methods: analysis of library quality) using a threshold of up to 10% substitutions in matching DNA region or a Smith – Waterman score of at least 2500. For initial selection we preferred not to use stringent criteria in order not to leave aside nuclear genes and to allow assembly of contigs, a process that would not succeed without this screening. This first screening allowed eliminating 4,631 mitochondrial clones. Analysis of the remaining clones revealed the presence of 911 additional mitochondrial sequences (E value smaller or equal to e-06), 521 singletons and 41 contigs. Out of a total of 5,542 sequences, 4869 matched with sequences located between nucleotides 9,450 and 11,700 of the mitochondrial genome. This region contains the large subunit ribosomal RNA and three tRNA genes. It is possible that these sequences allowed RNA:tRNA dimer formation and initiation of cDNA synthesis as described in some retroviruses [22]. The mitochondrial sequences could be used to study metabolism in microarray experiments. The number of sequences that are not mitochondrial or *Candidatus *Portiera aleyrodidarum from the various libraries is as follows: EGG: 357, HBT:1873, INST:1529, TOMOV:1985 and TYLCV:2367 (submitted to dbEST Genbank).

**Figure 3:**
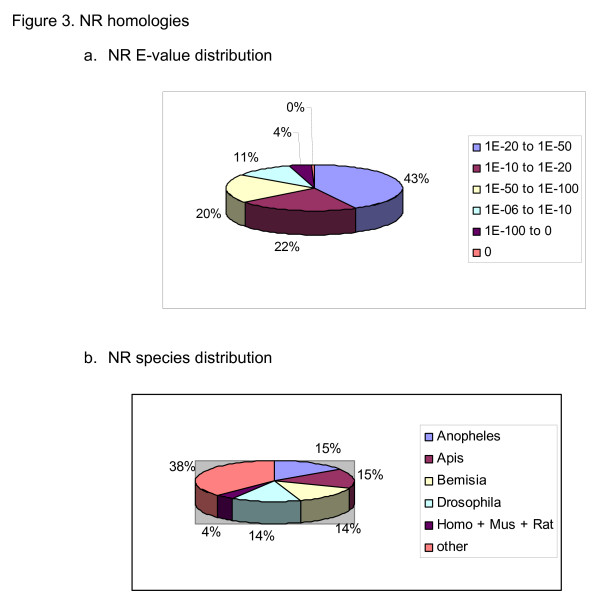
**NR-Homologies a. NR E-value distribution **Nr E-value distribution is shown as a percent of the total top homologies. Represented are the 1,544 contigs and singletons that had a homology to a protein in the nr database with an E-value of at least 1.0e-06. **b. NR species distribution **Species distribution is shown as a percent of the total top homologies. Represented are the 1,544 contigs and singletons that had a homology to a protein in the nr database with an E-value of at least 1.0e-06 (abbreviations used: Homo for *Homo sapiens*, Mus for *Mus musculus *and Rat for *Rattus norvegicus*).

The third largest contig shared high homology to other insect vitellogenin genes; this contig is also the second longest (2,883 bp) and originated exclusively from the adult whitefly libraries (Figure 2). Since the libraries were not normalized, a high level of redundancy allowed appraising the level of expression of certain genes at certain developmental stages, or as a consequence of virus ingestion and/or transmission. Figure 3 shows that in the thirteen longest contigs (each containing 28 or more sequences) the INST library seems to have a different set of expressed genes than the adult libraries. Contig Bt-ToMoV-023-1-C12-T3_C12 was composed of 28 sequences which shared no significant homology with any of the sequence databases that were searched. However a search of the Interpro database [23] has revealed that it contains a signal peptide and a *trans*-membrane domain.

### Identification of whitefly contigs and singletons by BLAST analysis

To identify homologies and identities of the contigs and singletons to known proteins, genes and/or genomes, contigs and singletons were subjected to blastn and blastx searches against the following databases: non-redundant protein database (nr, NCBI), non-redundant nucleotide database (nt, NCBI), Swiss-Prot [24], Flybase protein database [25] and EST other (non-human and non-mouse, NCBI) [see Additional file 3] Managing and parsing the BLAST outputs were carried out using the BioCloneDB application [26]. About 45% of the contigs and singletons had a match with an E value of at least 1.0e-06 to one of the above databases (Table 4). 1,544 contigs and singletons had a homology to a protein in the nr database. The E-value distributions of the top hits in the nr database (Figure 3a) showed that 43% of the homolog contigs and singletons ranged between 1.0e-20 to 1.0e-50, whereas 67% had a moderate to strong homology (smaller than 1.0e-20). The species distribution of the top hits (Figure 3b) showed that 58% of contigs and singletons had sequence homology to genomes of insects completely or partially sequenced, and were approximately evenly distributed between the mosquito *Anopheles gambiae*, the honeybee *Apis mellifera*, the whitefly *B. tabaci *and the fruitfly *D. melanogaster*.

**Table 4:** Number of contigs (out of the 4,860) that had a significant hit (E-value equal or smaller than 1.0e-06) with the listed databases

**Database**	**Number of contigs and singletons**
Drosophila	1,053
Nr	1,544
Nt	1,207
Swissprot	1,224
EST other	1,224
Contigs and singletons found in at least one of the above databases	2,211

### Sequences with no identifiable homology

No homologous sequences could be found for 2,649 (54.5%) of the contigs and singletons among the databases searched. The singletons showed a higher occurrence of lack of homology (58%). Because the library was poly(dT)-primed, some of these sequences may represent 3' untranslated regions (3' UTRs). It is also possible that the putative homologous regions are too short to produce a significant alignment.

### Comparison to existing *B. tabaci *sequences in NCBI

There were 448 contigs and singletons presenting high similarity (E-value equal or smaller than 1.e-40) with *B. tabaci *DNA sequences. The majority of these hits (399) were to the whitefly mitochondria genome (AY521257.1), and represented mitochondrial sequences that were not removed in the preassembly process. The BLAST search against the EST database did not reveal any ESTs originating from *B. tabaci *(currently there are no *B. tabaci *ESTs in Genbank). Thus, homology searches indicated that the majority of the contigs and singletons described herein are novel *B. tabaci *genes, which are not known in the NCBI sequence databases.

### Assignment of the whitefly contigs and singletons to common Gene Ontology terms

**Figure 4:**
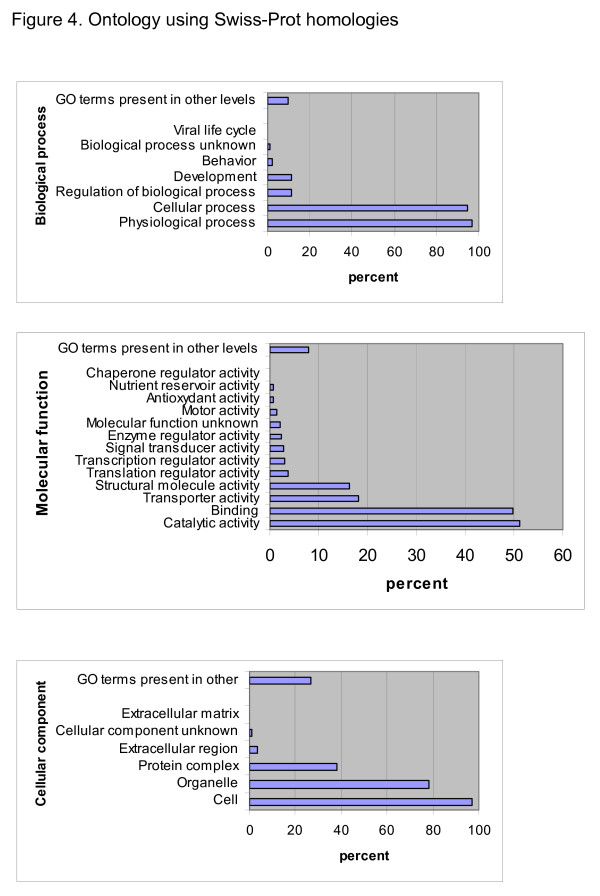
**Ontology using Swiss-Prot homologies **The Swiss-Prot homologs were used as a query for the FatiGO tool. The output of FatiGO is summarized here in three main categories in level 2: biological process, molecular function and cellular component.

Based on homologies with the Swiss-Prot database, the contigs and singletons were assigned a biological process, molecular function and cellular component from the Gene Ontology (GO) terminology [27]. The GO was extracted electronically using the FatiGO tool [28]. The top hit of 1,224 contigs and singletons with an E-value equal or smaller than 1.0e-06 was to 922 different Swiss-Prot entries (Figure 4). According to the FatiGo analysis on this list of Swiss-Prot entries only 35 did not have a GO annotation. The most dominant Biological process GO annotation at level 2 was physiological process (97% of the proteins) and 95% were annotated as cellular process. The most dominant molecular function GO category was catalytic activity (51%) and binding (50%) in level 2. The most dominant cellular component was cell (97%) and the second largest was organelle (78%).

### Comparing whitefly Gene Ontology to *Drosophila*

To evaluate how similar *B. tabaci *is to *Drosophila*, a blastx search was carried out against all *Drosophila *proteins. Here, 1,053 contigs and singletons had a top hit with an E-value equal, or smaller, than 1.0e-06. This set of genes was used to compare *B. tabaci *to *Drosophila *in respect to their GO profile. The overrepresented terms were associated with ribosome and protein biosynthesis as well as mitochondria and generation of precursor metabolites and energy (Table 5). The underrepresented terms were 'unknowns', or were identified as receptors, or as having a role in signal transduction (Table 6).

**Table 5:** *Drosophila *homologs used to discover over-represented ontologies in the *B. tabaci *contigs and singletons

**Best GOs**	**Term**	**Count**	**Total**	**P-Value**
(Max: 50)		732	10,309	
GO:0005840	Ribosome	106	202	0
GO:0005737	cytoplasm	357	1,800	0
GO:0030529	ribonucleoprotein complex	124	370	0
GO:0005830	cytosolic ribosome (sensu Eukaryota)	74	98	0
GO:0003735	structural constituent of ribosome	106	194	0
GO:0043234	protein complex	306	1623	0
GO:0015934	large ribosomal subunit	58	103	6.50E-81
GO:0015935	small ribosomal subunit	44	74	3.73E-65
GO:0005829	Cytosol	122	440	2.52E-63
GO:0005739	mitochondrion	131	509	2.87E-60
GO:0005622	intracellular	467	3,678	2.12E-58
GO:0044249	cellular biosynthesis	205	1,077	1.06E-55
GO:0043229	intracellular organelle	394	2,919	2.21E-54
GO:0043226	Organelle	394	2,919	2.21E-54
GO:0043228	non-membrane-bound organelle	160	744	3.87E-54
GO:0043232	intracellular non-membrane-bound organelle	160	744	3.87E-54
GO:0044237	cellular metabolism	562	5,047	1.27E-52
GO:0009058	biosynthesis	208	1,145	2.01E-51
GO:0008152	metabolism	584	5,438	7.70E-50
GO:0006412	protein biosynthesis	151	731	4.65E-47
GO:0009059	macromolecule biosynthesis	154	760	2.74E-46
GO:0005743	mitochondrial inner membrane	64	187	3.25E-45
GO:0019866	inner membrane	64	187	3.25E-45
GO:0006119	oxidative phosphorylation	56	152	6.10E-44
GO:0005746	mitochondrial electron transport chain	38	82	1.29E-40
GO:0005740	mitochondrial membrane	68	222	1.37E-40
GO:0042773	ATP synthesis coupled electron transport	35	72	8.45E-40
GO:0050875	cellular physiological process	645	6,729	3.39E-39
GO:0005759	mitochondrial matrix	52	151	7.69E-37
GO:0005842	cytosolic large ribosomal subunit (sensu Eukaryota)	42	57	1.12E-34
GO:0043170	macromolecule metabolism	354	2,902	2.19E-34
GO:0005198	Structural molecule activity	140	760	2.73E-34
GO:0044238	primary metabolism	512	4,908	4.18E-34
GO:0016282	eukaryotic 43S preinitiation complex	43	64	8.21E-33
GO:0044260	cellular macromolecule metabolism	330	2,676	1.98E-32
GO:0015077	monovalent inorganic cation transporter activity	49	151	1.09E-31
GO:0009987	cellular process	661	7,297	2.03E-31
GO:0015078	Hydrogen ion transporter activity	48	149	1.10E-30
GO:0007582	physiological process	674	7,566	1.67E-30
GO:0005623	Cell	512	5,045	3.62E-30
GO:0006091	generation of precursor metabolites and energy	106	532	4.41E-30
GO:0031090	Organelle membrane	82	382	1.28E-26
GO:0005843	cytosolic small ribosomal subunit (sensu Eukaryota)	32	43	1.28E-26
GO:0016283	eukaryotic 48S initiation complex	32	43	1.28E-26
GO:0044267	cellular protein metabolism	293	2,433	1.48E-25
GO:0015399	primary active transporter activity	52	192	3.21E-25
GO:0019538	protein metabolism	293	2,452	7.25E-25
GO:0043231	intracellular membrane-bound organelle	291	2,524	1.50E-21
GO:0043227	membrane-bound organelle	291	2,524	1.50E-21
GO:0005761	mitochondrial ribosome	28	76	1.69E-21
	Ribosome mitochondrial andmetabolism enriched			

**Table 6:** *Drosophila *homologs used to discover under-represented ontologies in the *B. tabaci *contigs and singletons

**Best GOs**		**Count**	**Total**	**P-Value**
		732	10309	
GO:0005554	molecular_function unknown	8	939	3.31E-13
GO:0000004	biological_process unknown	8	810	9.08E-11
GO:0008372	cellular_component unknown	17	983	2.48E-10
GO:0004871	signal transducer activity	39	1,092	3.28E-05
GO:0004872	receptor activity	14	584	0.000109
GO:0004888	transmembrane receptor activity	9	452	0.000286
GO:0007165	signal transduction	58	1,303	0.000994

### Mapping whitefly contigs and singletons to pathways

From the top nr homologies the additional information extracted are the KEGG EC numbers [29]. In total, out of the 1,544 nr hits, 48 had an EC number, 37 of which were unique. The EC numbers were mapped to their respective pathway using the KEGG tools (gpath) (Table 7).

**Table 7:** Mapping the pathways for the 37 unique EC numbers extracted by the BiocloneDB application for the nr homologs. The output was produced by the 'KEGG gpath tool'.

**Pathway**	**EC accession**
map00010 Glycolysis / Gluconeogenesis	EC 1.2.1.3
	EC 3.1.3.
	EC 5.3.1.
map00030 Pentose phosphate pathway	EC 1.1.1.44
	EC 1.1.99.10
	EC 3.1.3.11
map00031 Inositol metabolism	EC 5.3.1.1
map00040 Pentose and glucuronate interconversions	EC 2.4.1.17
map00051 Fructose and mannose metabolism	EC 3.1.3.11
	EC 5.3.1.1
map00052 Galactose metabolism	EC 2.7.7.12
map00053 Ascorbate and aldarate metabolism	EC 1.2.1.3
map00071 Fatty acid metabolism	EC 1.2.1.3
map00120 Bile acid biosynthesis	EC 1.2.1.3
	EC 1.2.1.3
map00130 Ubiquinone biosynthesis	EC 1.6.5.3
map00150 Androgen and estrogen metabolism	EC 2.4.1.17
map00190 Oxidative phosphorylation	EC 1.6.5.3
	EC 1.9.3.1
	EC 1.10.2.2
	EC 3.6.3.14
map00193 ATP synthesis	EC 3.6.3.14
map00195 Photosynthesis	EC 3.6.3.14
map00220 Urea cycle and metabolism of amino groups	EC 2.6.1.11
	EC 6.3.4.5
map00230 Purine metabolism	EC 2.7.4.6
	EC 2.7.7.6
map00240 Pyrimidine metabolism	EC 2.7.4.6
	EC 2.7.4.9
	EC 2.7.7.6
map00251 Glutamate metabolism	EC 6.3.1.2
map00252 Alanine and aspartate metabolism	EC 6.3.4.5
map00260 Glycine, serine and threonine metabolism	EC 1.1.1.95
	EC 2.3.1.37
	EC 2.7.1.32
map00280 Valine, leucine and isoleucine degradation	EC 1.2.1.3
map00310 Lysine degradation	EC 1.2.1.3
map00330 Arginine and proline metabolism	EC 1.2.1.3
	EC 6.3.4.5
map00340 Histidine metabolism	EC 1.2.1.3
map00380 Tryptophan metabolism	EC 1.2.1.3
map00400 Phenylalanine, tyrosine and tryptophan biosynthesis	EC 4.2.3.4
	EC 6.1.1.20
map00410 beta-Alanine metabolism	EC 1.2.1.3
map00480 Glutathione metabolism	EC 2.5.1.18
map00500 Starch and sucrose metabolism	EC 2.4.1.1
	EC 2.4.1.17
map00520 Nucleotide sugars metabolism	EC 2.7.7.12
map00530 Aminosugars metabolism	EC 3.2.1.14
map00550 Peptidoglycan biosynthesis	EC 6.3.1.2
map00561 Glycerolipid metabolism	EC 1.2.1.3
map00564 Glycerophospholipid metabolism	EC 2.7.1.32
map00620 Pyruvate metabolism	EC 1.2.1.3
	EC 2.7.9.2
map00630 Glyoxylate and dicarboxylate metabolism	EC 4.1.1.39
map00631 1,2-Dichloroethane degradation	EC 1.2.1.3
map00640 Propanoate metabolism	EC 1.2.1.3
map00650 Butanoate metabolism	EC 1.2.1.3
map00710 Carbon fixation	EC 3.1.3.11
map00720 Reductive carboxylate cycle (CO2 fixation)	EC 2.7.9.2
map00860 Porphyrin and chlorophyll metabolism	EC 2.4.1.17
	EC 4.99.1.1
map00903 Limonene and pinene degradation	EC 1.2.1.3
map00910 Nitrogen metabolism	EC 6.3.1.2
map00970 Aminoacyl-tRNA biosynthesis	EC 6.1.1.20
map02040 Flagellar assembly	EC 3.6.3.14
map03020 RNA polymerase	EC 2.7.7.6
map03050 Proteasome	EC 3.4.25.1
map03070 Type III secretion system	EC 3.6.3.14

### Multiple alignments of vitellogenin-like contigs

**Figure 5:**
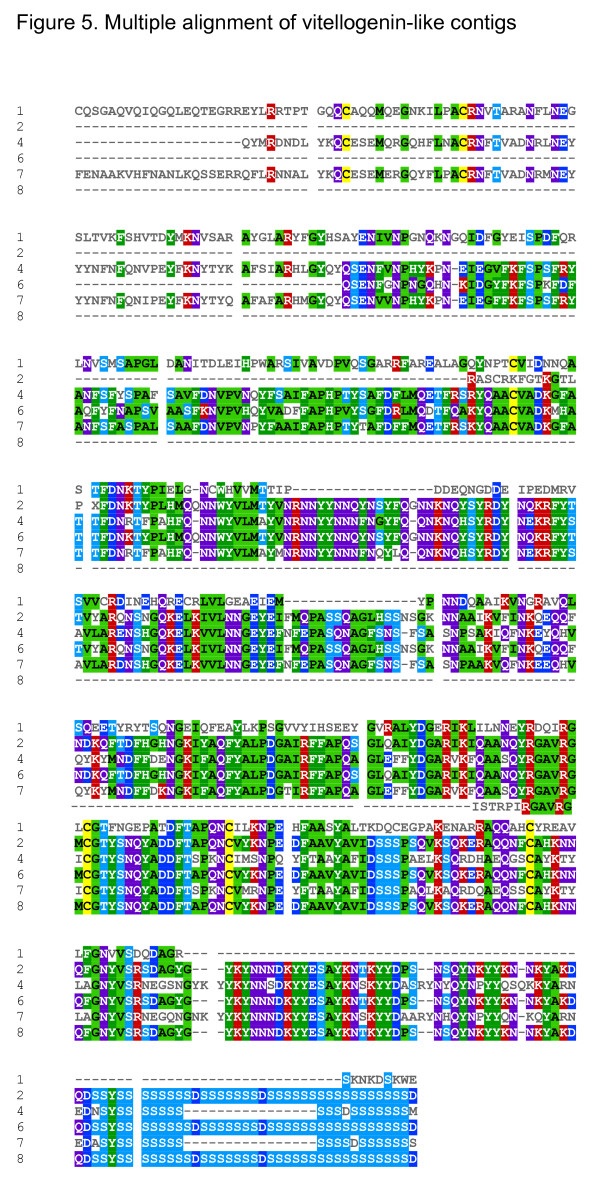
**Multiple alignment of vitellogenin-like contig **Protein multiple alignments of five contigs with BAA22791.1 (amino acid 1371 to 1770) used as a profile. The numbers in the figure designate the following sequences :(1) BAA22791.1 (2) BT-TOMOV-020-1-D2-T3_D02 (4) BT-TYLCV-030-1-C9-T3_C09 (6) BT-TYLCV-043-1-D2-T3_D02 (7) BT_TYLCV004_B07 (8) TMVBT002_D07. Mview parameters: Identities computed with respect to: (1) gi_2522237_dbj_BAA22791.1 and coloured by: consensus/50% and property

**Figure 6:**
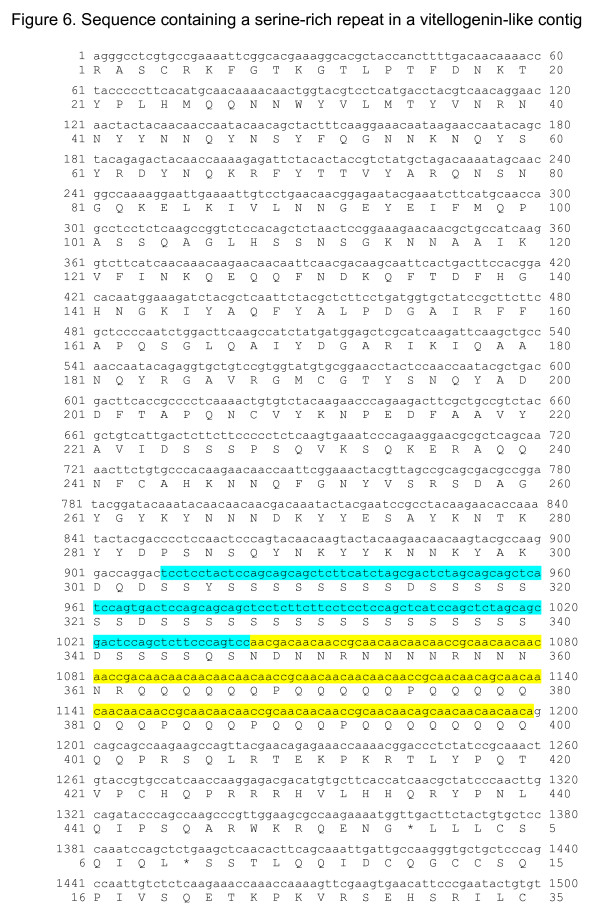
**Repeats in the vitellogenin-like contig **Translation of BT-TOMOV-020-1-D2-T3_D02 from 1 to 1611. The region of the AAC repeats is highlighted in yellow. The region rich in serine codons is highlighted in blue.

Nine contigs shared the same vitellogenin homolog from the butterfly *Athalia rosae*; (BAA22791.1) as revealed in a blastx search against nr-database. Table 8 shows homologies to vitellogenin ranging from an E-value of 6.0e-13 to 2.0e-83; the location of homology was not the same for the various contigs. To evaluate whether the contigs encoded a protein family or the same protein, a multiple alignment of the contigs having homology to the carboxyl terminus of vitellogenin (amino acids 1371 to 1770, BAA22791.1) was performed (Figure 5). The multiple alignments clearly demonstrated that at least two different types of proteins were represented (2, 6 and 8 versus 4 and 7). The contigs shared a region rich in serine codons flanked by an AAC repeat in the DNA sequence, two features not found in the C-terminal moiety of the *A. rosae *vitellogenin homolog (Figure 6). In *A. rosae *vitellogenin, two serine repeats can be found in the N-terminal moiety of the protein, between amino acids 344 and 367 and between amino acids 402 and 434.

**Table 8:** Results of nine contigs BLAST searched against gi|2522237|dbj|BAA22791.1| vitellogenin from the butterfly *Athalia rosae*

**Contig Name**	**% identity**	**Alignment length**	**Contig start**	**Contig end**	**Vitellogenin start**	**Vitellogenin end**	**E-value**
BT-TYLCV-021-1-E5-T3_E05_1	50.72	207	618	1	1	207	9.20E-60
BT-TOMOV-035-1-E6-T3_E06_1	58.33	84	595	344	241	324	4.10E-25
BT-TYLCV-039-1-F7-T3_F07_1	46.97	132	398	3	478	607	3.90E-31
BT_TYLCV004_B07_1	30.97	649	5	1,885	1,141	1,755	9.80E-89
BT-TYLCV-024-1-F5-T3_F05_1	39.02	123	372	4	1,256	1,378	3.60E-20
BT-TYLCV-030-1-C9-T3_C09_1	30.3	396	3	1,187	1,391	1,755	6.80E-54
BT-TYLCV-043-1-D2-T3_D02_1	34.14	331	8	997	1,463	1,759	2.90E-49
BT-TOMOV-020-1-D2-T3_D02_1	35.25	244	46	777	1,550	1,759	1.50E-35
TMVBT002_D07_T3_061_1	48.68	76	20	247	1,684	1,759	2.80E-18

### The whitefly contig and EST database

A relational database with a web-based front end (WhiteFlyDB) was created to store, navigate, annotate and retrieve sequence and contig information [30]. This database is based on the BioCloneDB application [26]. The database contains all the relevant contig information such as the names of the sequence that compose it, the top hits against the described databases and the information extracted for these top hits (GO, EC, cellular location) as well as information on the homology itself. The sequences in fasta format and the tab delimited BLAST reports can be easily extracted and imported to Excel files.

## Discussion

The whitefly *B. tabaci *is a major pest to agricultural crops because it causes damage due to feeding and because it transmits many important viruses to plant species cultivated for food and fiber nearly worldwide. Previous to the present research and despite the importance of *B. tabaci*, the sequence of only a handful of mRNAs (mostly partial) encoding a handful of nuclear protein-coding genes has been published in Genbank. They include sequences encoding actins, a para-sodium channel, putative knottins, a NADP-dependent ketose reductase, two heat shock proteins, a nicotinic acetylcholine receptor alpha subunit, an acetyl cholinesterase-like protein, and a diffusible secreted glycoprotein. The results described in this communication represent the first attempt to develop a functional genomics program involving a homopteran species.

Since the amount of total RNA that could be extracted from eggs and instars was extremely low, we have not isolated polyA^+^-RNA, which has inevitably reduced the mRNA representation in the sample. Instead we have used total RNA as template for synthesizing cDNA. Libraries have been prepared from another insect pest, the brown citrus aphid *Toxoptera citricida *starting from RNA samples enriched in polyA^+^-RNA [31]. However, it has to be noted that an adult aphid weighs approximately 300 micrograms, while an adult whitefly weighs approximately 30 micrograms. Moreover, the weight of a whitefly egg is approximately 1/1000 that of an adult. We have not normalized the libraries, a fact that allowed us to roughly estimate and compare the levels of expression of major genes in the different libraries.

The fraction of the expressed whitefly genes present in our database can be roughly estimated. Although the genome size of *B. tabaci *was estimated to be approximately five times that of *Drosophila *[19], it is logical to speculate that the two insect species may have approximately the same number of protein-encoding nuclear genes. The whitefly database contains the sequences of 975 contigs and 3,322 singletons (non-mitochondrial and non-bacterial). If we take into account that each contig represents a transcript of a single protein-coding nuclear gene our sequences represent 4,297 genes. The number of gene families (protein families) in *Drosophila *has been estimated as 674 and the number of genes not member of a gene family has been estimated as 10,786; altogether 11,460 protein-encoding genes [32]. Hence the *B. tabaci *database may represent approximately one third of the insect nuclear protein-encoding genes. Additional sequencing from the 3' end of the clones may provide a more accurate estimation.

Within this whitefly database approximately half of the sequences had a match with an E value of at least 1.0e-06 to one of the databases; 1,544 sequences had a homology to a protein in the nr database. Approximately 60% of the whitefly contigs presented homologies with sequenced genomes of other insect species. No homologous sequence could be found for 2,649 contigs and singletons (54.5%) with any of the databases searched.

It was notable that the most abundant contig was vitellogenin. This ancient protein is the major yolk protein of eggs, where it is used as a food source during embryogenesis [33]. There are three vitellogenin genes in *Drosophila *[25]. The whitefly vitellogenin sequences were found exclusively in libraries from adult whiteflies, indicating that a relatively large amount of resources transcriptional activity is mobilized towards the production of eggs.

The database developed in this study provides a large source of information for studies of whitefly development, circulative transmission of begomoviruses, and choice of host plant. Comparing the sequences present in the various libraries may provide preliminary information on genes expressed during acquisition and transmission of begomoviruses, and ultimately those involved in *B. tabaci *development.

## Conclusion

The set of sequences developed in this study makes available the first DNA sequence database for an important hemipteran (homopteran) pest of agricultural crops for the scientific community. Its availability will allow the investigation of important questions regarding whitefly biology, development, gene expression, and comparative biology. It will also facilitate studies to elucidate the genetics underlying gene expression in pest- and non-pest biotypes, and the basis for virus-vector specificity, resistance to insecticides, and plant host preferences for this cryptic species. This sequence set has been arrayed in a microchip format and enables biologically-based questions to be addressed by examining gene functionalities and expression patterns of the whitefly genome.

## Methods

### Libraries from adult viruliferous and non-viruliferous whiteflies

Directional cDNA libraries were constructed in the Lambda Uni-ZAP^® ^XR vector using 5 μg of total RNA according to the manufacturers' instructions (Stratagene, La Jolla, CA). Whiteflies (*B. tabaci*, B biotype) were reared on TYLCV-infected tomato plants, on ToMoV-infected tomato plants and on non-infected tomato plants. The plants were kept in insect-proof cages. Insects were collected randomly from each of the colonies and placed at -80°C. Total RNA was isolated from pools of approximately 200 insects using the RNeasy Midi kit (QIAGEN). Contaminating DNA was discarded using RNAase-free DNAse I. Double stranded cDNA was synthesized using the cDNA Synthesis Kit (Stratagene) with primers containing each *Eco*RI and *Xho*I sites. Blunt ends were created, ligated to *Eco*RI adaptors, phosphorylated and digested with *Eco*RI. The cDNAs were size-fractionated using Drip Columns to obtain molecules longer than 300 bp. The cDNA was ligated to the Zap Express vector and used to transform *E. coli *cells in the presence of kanamycin. The phagemid pBK-CMV was packaged using the Gigapack III Gold packaging (Stratagene). Phages were used to inoculate XL1-Blue MRF cells. A library was generated with a titer of 5.2 × 10^10 ^plaque-forming units per ml. The library was mass excised using Ex-Assist^® ^helper phage (Stratagene) and bacterial clones containing excised pBluescript SK+ phagemids were recovered by random colony selection.

### Libraries from eggs and instars

Directional cDNA libraries were constructed using the Creator SMART cDNA Library Construction Kit (Clontech). Eggs and instars were collected from leaves of cotton plants caged with whiteflies (*B. tabaci*, B biotype) and held in insect-proof cages. RNA was isolated using TRIZOL and Phase Lock Gel-Heavy tubes. The first strand cDNA was synthesized using the total RNA and the CDS III/3' PCR Primer which contains a *Sfi *IB site. The cDNA was amplified by PCR: the first-strand cDNA was used together with the 5' PCR primer which contains a *Sfi *IA site and the CDS III/3' PCR primer. Following phenol treatment, the DNA was digested with *Sfi*I and size-fractionated using CHROMA SPIN-400 Column. The high molecular weight cDNA fractions (with sticky ends) were pooled together and ligated with dephosphorylated pDNR-LIB vector treated *Sfi *IA and *Sfi *IB. The recombinant plasmids were electroporated into DH5α and 10G competent cells, and plated on LB agar plate containing chloramphenicol.

### Sequencing

Plasmid clones were isolated from 1.7 ml overnight Luria-Bertani broth cultures using a Qiagen 9600 robot and Qiaprep 96 turbo plasmid isolation kits (Qiagen, Valencia, CA). Plasmid DNA (80 ng) was used as a template for ABI Prism^® ^BigDye™ terminator cycle sequencing (PE Applied Biosystems, Foster City, CA.). Sequencing of the adult whitefly libraries was from the 5' end of the cloned cDNA using a T3 promoter universal primer: 5'ATTAACCCTCACTAAAGGGA3'. Sequencing of the egg and instar whitefly libraries was from the 5' end of the cloned cDNA using a M13 primer: 5'GAAGTTATCAGTCGACGG-3'. Reactions were concentrated and washed by ethanol precipitation. Pellets were resuspended in 15 μl of formamide prior to separation on an ABI Prism 3700 Sequencer (USHRL/ARS/USDA Genomics Laboratory, Fort Pierce FL).

### Analysis of library quality

Mitochondrial sequences were not considered for clustering. Significant homology to the *B. tabaci *mitochondrion, complete genome (NC_006279.1) was found using cross_match [34]. Of the 18,900 sequences analyzed 5,542 were found to contain significant mitochondrial sequences. However, as further analysis revealed the sequences that passed this filtering still contained some mitochondrial contamination.

### Analysis and assembly of sequence data

Analysis of the chromatograms was done using Staden pregap4 [20] and the following integrated programs: Phred, cross_match and RepeatMasker [34]. Passed sequences were required to be longer than 75 bases after the trimming procedure. Cross_match and RepeatMasker were used to detect additional vector and adapter contamination after the sequence vector clip. A script was written for trimming the vector from the sequence ends, according to the information obtained by cross_match. Assembly of the contigs was carried out using the Staden gap4 normal assembly feature. Assembled contigs were used to perform BLAST searches (BLASTX and BLASTN) locally using the NCBI-BLAST [35] against the non-redundant protein sequence database, *Drosophila *proteins (dmel-translation), Swiss-Prot and EST_other (released March-May 2005), which were downloaded from the NCBI [36] or the FlyBase [25] databases.

The database application BiocloneDB [26] was used to manage the BLAST run, and to parse homologue alignment information, whereby an E-value of 1e-06 was used as a maximum cut-off. This information was stored in the SQL database. The DB application also supports sequence and contig queries and down-loads through the web interface [30].

### Electronic annotation of contigs and singletons

The BiocloneDB application was used to extract from the EBI SRS information regarding the closest annotated homolog of contigs and singletons, including the GO annotation [27], EC number [29] and cellular location. The ontology distribution, according to the Swiss-Prot homologous proteins, was determined using the FatiGO tool [28]. KEGG tool [37] was used to find the pathway distribution with the nr homologies, which contain an EC number. Over- and under-represented ontologies, with respect to the *Drosophila *genome, were *Drosophila melanogaster *homologies using the GOstat tool [38].

### Multiple alignment

Multiple sequence alignment of the 5 translated contigs which their best homology was to Vitellogenin precursor from *A. rosae *(BAA22791.1) and their homology was to the same region in the *A. rosae *protein ranging from amino acid 1400-1920. The alignment was done by VectorNTI AlignX tool using the *A. rosae *protein as a profile. The MSF file produced is displayed using the Mview tool [39].

### Other bioinformatic tools

Translation view was accomplished using the Prettyseq tool and percent GC calculations were carried out using Geecee. Both tools are part of the EMBOSS suite [40].

## Authors' contributions

HC, CM, RLS, JKB, overall project supervision; SG, MG, JKB, preparation of libraries; CM, RLS, sequencing of clones; DL, ER, sequence processing, assembly, annotation and bioinformatic analyses; ER, DL, development of whitefly database search engine; DL, HC, CM, RLS, JKB, preparation of manuscript; ER, JKB preparation of web site.

## Supplementary Material

Additional File 1 This file contains sequence information regarding the sequences and the contigs they assembled. Click here for file

Additional File 2 This file contains contigs and singletons information such as a list of sequence names assembling the contig and their library count, contig length, GC content and more. Click here for file

Additional File 3 This file contains the top BLAST hit for each contig and singleton against the databases searched. Click here for file
